# Aesthetic Emotions Across Arts: A Comparison Between Painting and Music

**DOI:** 10.3389/fpsyg.2015.01951

**Published:** 2016-01-05

**Authors:** Andrei C. Miu, Simina Pițur, Aurora Szentágotai-Tătar

**Affiliations:** ^1^Cognitive Neuroscience Laboratory, Department of Psychology, Babeş-Bolyai UniversityCluj-Napoca, Romania; ^2^Department of Clinical Psychology and Psychotherapy, Babeş-Bolyai UniversityCluj-Napoca, Romania

**Keywords:** aesthetic emotions, painting, music, art education

## Abstract

Emotional responses to art have long been subject of debate, but only recently have they started to be investigated in affective science. The aim of this study was to compare perceptions regarding frequency of aesthetic emotions, contributing factors, and motivation which characterize the experiences of looking at painting and listening to music. Parallel surveys were filled in online by participants (*N* = 971) interested in music and painting. By comparing self-reported characteristics of these experiences, this study found that compared to listening to music, looking at painting was associated with increased frequency of wonder and decreased frequencies of joyful activation and power. In addition to increased vitality, as reflected by the latter two emotions, listening to music was also more frequently associated with emotions such as tenderness, nostalgia, peacefulness, and sadness. Compared to painting-related emotions, music-related emotions were perceived as more similar to emotions in other everyday life situations. Participants reported that stimulus features and previous knowledge made more important contributions to emotional responses to painting, whereas prior mood, physical context and the presence of other people were considered more important in relation to emotional responses to music. Self-education motivation was more frequently associated with looking at painting, whereas mood repair and keeping company motivations were reported more frequently in relation to listening to music. Participants with visual arts education reported increased vitality-related emotions in their experience of looking at painting. In contrast, no relation was found between music education and emotional responses to music. These findings offer a more general perspective on aesthetic emotions and encourage integrative research linking different types of aesthetic experience.

## Introduction

Emotional responses to art (i.e., aesthetic emotions) have long interested philosophers, psychologists, and art critics (Robinson, 2004). Theories in psychology and aesthetics (James, 1890/1950; Bell, 1914; Berlyne, 1974) initially focused on positive emotional responses that arise from the appreciation of the form of expression as beautiful, harmonious, or powerful (Robinson, 2004). Recent studies have found that indeed, emotions (i.e., brief affective states triggered by the appraisal of an event in relation to current goals; Scherer and Zentner, 2001) such as awe (Shiota et al., 2007) and wonder (Zentner et al., 2008) are frequently reported in relation to the contemplation of artworks. These emotions typically occur when an object or event is appraised as highly complex and novel, and creates a sense of being in the presence of something greater than oneself (Keltner and Haidt, 2003).

However, it has also been recently emphasized that affective responses to art are more diverse (Silvia, 2011) and often include emotions such as sadness (Vuoskoski and Eerola, 2012) and nostalgia (Barrett et al., 2010), which are also experienced in other everyday situations that do not involve contemplation of artworks. These emotions may be related to the content and personal interpretation of an artwork, rather than its form (Robinson, 2004; Silvia, 2011). For instance, one may admire Caravaggio's skill in *David with the Head of Goliath*, but also feel disgust at the sight of dripping blood, and sadness at the thought that this artwork may express the painter's remorse. Similarly, someone listening to the *Adagietto* from Mahler's 5th Symphony may feel blends of awe, tenderness and nostalgia related to the skillful orchestration, on the one hand, and knowing that this piece captures the composer's love for his wife and worries for his deteriorating health, on the other hand. Therefore, art contemplation can trigger multiple emotions, which include aesthetic emotions driven by positive appraisals of the form of expression, and other positive or negative emotions, driven by appraisals of the content or meaning of artworks (Silvia, 2011). Given the increasing interest in affective science (Gross and Barrett, 2013), recent studies have focused on describing emotions associated with aesthetic experiences such as looking at painting and listening to music, and on examining their underlying mechanisms and motivation (for review see Silvia, 2011; Swaminathan and Schellenberg, 2015).

Influential theoretical frameworks, which have guided research on preferences for painting (Leder et al., 2004; Lindell and Mueller, 2011) and emotional responses to music (Scherer and Zentner, 2001), argue that one's reactions to artworks involve an interplay of multiple factors related to stimulus, person, and situation. The contribution of perceptual features and formal characteristics conveying style has been pointed out by observations that aesthetic preferences form very rapidly (i.e., in less than 1 s), whether in the form of beauty judgments of graphic patterns (Jacobsen and Höfel, 2003) or emotional categorization of music excerpts (Bigand et al., 2005). Indeed, these rapid responses may involve automatic mechanisms such as visual disambiguation (Topolinski et al., 2015) and premotor simulation (Leder et al., 2012; Ticini et al., 2014), although recent studies also report their interaction with consciously controlled processes such as expectations (McLean et al., 2015). The relations between the structural characteristics of music (e.g., mode, tempo) and emotional responses have been systematically investigated (Gabrielsson and Lindstrom, 2001; Gomez and Danuser, 2007). Taking a more general approach, research relevant to painting has mostly focused on non-aesthetic stimuli (e.g., geometrical shapes) and broad aesthetic preferences instead of specific emotions (Jacobsen and Höfel, 2002). Nonetheless, theory in both fields (Scherer and Zentner, 2001; Lindell and Mueller, 2011) has acknowledged that stimulus-driven or “bottom-up” processing interacts with education and psychological characteristics that can influence emotional responses to art through knowledge-driven or “top-down” processing.

Many studies have therefore examined whether art education facilitates art-related emotions through a better understanding of the formal means of expression in painting or music. Indeed, students in art history compared to students in other fields categorize paintings using more criteria and favor style-related rather than affective criteria (Augustin and Leder, 2006). Similarly, musicians perceive the links between a musical theme and its variations better than non-musicians (Bigand and Poulin-Charronnat, 2006), and describe music using adjectives related to novelty and originality rather than emotional characteristics (Istok et al., 2009). However, despite these differences in processing styles, music-related emotions are not markedly dissimilar in musicians and non-musicians (Bigand and Poulin-Charronnat, 2006; Baltes et al., 2012) and the same may be true for painting-related emotions. While no study investigated the influence of visual arts expertise on emotional responses to painting, experimental evidence suggests that providing additional information that facilitates understanding of paintings does not influence preference for paintings (Leder et al., 2006). In addition to art education, other individual differences such as prior mood may also influence emotional responses to artworks (Hunter et al., 2011; Vuoskoski and Eerola, 2011a,b; Baltes and Miu, 2014).

Situational factors may also modulate art-related emotions. For instance, the presence of other people such as in the attendance of live music performance or during a visit to an art gallery may influence emotional responses to artworks. Field studies (Juslin et al., 2008) and experimental studies (Liljestrom et al., 2013) showed that the presence of the romantic partner or a close friend during music listening increases the frequency of affective states such as happiness-elation, pleasure-enjoyment and admiration-awe. These findings highlight social facilitation as one of the factors that may contribute to the increased enjoyment of music during live music performance (Lamont, 2011). The influence of context has also been acknowledged in theories of painting-related emotions (Leder et al., 2004) and one study (Pelowski et al., 2014) suggested that social encounters in art galleries may be detrimental to aesthetic experience by inducing competition between social awareness and self-focused enjoyment of paintings. However, the influence of social factors and other contextual variables (e.g., location; Scherer and Zentner, 2001) needs further research, particularly in the case of painting-related emotions.

In addition to mechanisms, recent studies have also focused on motivation for exposure to art. The most commonly reported reason for music listening is “mood repair” or emotion regulation, but social reasons (e.g., alleviate loneliness; keep up with art trends) and self-actualization needs (e.g., explore and express identity) are also frequently reported (Lonsdale and North, 2011). People use music to manage their mood to a greater extent than they use other leisure activities such as reading or exercising (Lonsdale and North, 2011). However, the tendency to use music for mood repair may be influenced by music training considering that musicians use music for cognitive (e.g., attention to structural complexity or performing technique) rather than emotional reasons (Getz et al., 2014). To our knowledge, no study has yet investigated motivation for aesthetic experience with painting.

In summary, painting and music-related emotions seem to involve a similar interplay of factors related to stimulus, person and context. However, any attempt to generalize across experience with these arts is currently hampered by the lack of empirical evidence on certain issues, particularly in the case of painting (e.g., frequency of specific emotions; influence of visual arts expertise, prior mood and social context; motivation), as well as the absence of integrative studies systematically comparing the characteristics of aesthetic experience in relation to painting and music (but see Rawlings et al., 2000; Cleridou and Furnham, 2014). In this study, parallel surveys on the experience of looking at painting and listening to music were filled in online by two samples of volunteers. Self-reported frequency of emotions, evaluation of contributing factors, and motivation in aesthetic experience with painting and music were compared between samples. In addition, the influence of art education on the characteristics of aesthetic experience was also investigated.

## Material and methods

### Participants

The surveys on looking at painting and listening to music were separately advertised online, mainly through social media (e.g., Facebook), as part of a psychological study on aesthetic experience. The survey on looking at painting was filled in by 260 participants, and the survey on listening to music was filled in by 711 participants. The surveys were in Romanian and all participants reported Romanian as their first language. Table 1 shows the distributions of age, sex, general education, and occupational status, which were not significantly different between the samples. Participants were informed that they would answer questions about their experience of looking at painting or listening to music, and signed a consent form before accessing the survey. The study followed the recommendations of the Declaration of Helsinki regarding participant safety and was approved by the Ethics Committee of Babeş-Bolyai University.

**Table 1:** **Socio-demographic characteristics of the survey samples**.

	**Age (M ± SD)**	**Sex (%)**		**General education (%)**			**Occupational status (%)**			
		**Women**	**Men**	**Primary**	**Secondary**	**Higher**	**Student**	**Employed**	**Unemployed**	**Retired**
Looking at painting	30.27 ± 11.53	82.31	17.69	5	30.38	64.62	40.77	52.31	4.23	2.69
Listening to music	28.10 ± 10.17	78.62	21.38	3.94	36.71	59.35	45.15	49.37	3.52	1.96

*Abbreviations: M, mean; SD, standard deviation*.

### Surveys

The questions and answer options were equivalent in the two surveys. Other than the reference to painting or music, the phrasing was identical.

The surveys were divided into three sections. The first section focused on socio-demographic characteristics: age, sex, education level, and occupational status.

The second section surveyed art education, asking participants whether they had graduated from a high school or college in the field of visual arts or music. Participants who filled in the survey on painting-related experiences were also asked to report whether they had knowledge related to painting or drawing, sculpture, and/or art history. Those who filled in the survey on music-related experiences were asked to report whether they had knowledge related to sight reading of musical scores, instrument playing and/or musicology. They were also asked to assess how experienced they thought they were in looking at painting or listening to music (five-point scale: 1, beginner; 5, experienced), as well as the personal importance of these art-related activities (five-point scale: 1, not at all important; 5, very important).

The third section included questions about frequency of art-related emotions, perception of contributing factors, and motivation for aesthetic experience. Emotional experience was assessed by asking participants to rate the frequency of several emotions in relation to looking at painting or listening to music, using a five-point scale (1, never; 2, rarely; 3, sometimes; 4, frequently; 5, very frequently). The emotion labels were taken from the 25-item version of the Geneva Emotional Music Scale (Zentner et al., 2008), representing nine emotion categories: wonder, transcendence, tenderness, nostalgia and peacefulness (facets of the more general dimension of “sublimity”); power and joyful activation (facets of “vitality”); and tension and sadness (facets of “unease”). To our knowledge, GEMS is the only standardized instrument covering the whole spectrum of emotional responses to artworks, including both positive aesthetic emotions (e.g., wonder, transcendence), and other positive (e.g., joyful activation, power) and negative emotions (e.g., nostalgia, sadness) that occur in various situations in everyday life. There is no equivalent standardized assessment of emotional responses to painting and developing such an instrument was beyond the purpose of this study. However, we thought GEMS was suitable for this exploratory study considering the potential similarities between emotional responses to music (Zentner and Eerola, 2010) and painting (Silvia, 2011). The Romanian translation of GEMS was used in several previous studies (e.g., Miu and Baltes, 2012; Baltes and Miu, 2014).

In addition to assessing the frequency of emotions using GEMS, another item asked participants to rate the similarity between everyday emotions and emotional experience with painting or music using a five-point scale (1, not at all; 5, very much).

Participants also rated, on a scale from 1 (not at all) to 5 (very much), the extent to which painting or music-related emotions involved one of the following factors: (1) structural features of the aesthetic stimulus, such as form, color, contrasts and composition for painting, and mode and tempo for music; (2) physical context (e.g., location); (3) prior mood, immediately before exposure to artworks; (4) previous knowledge about artwork and artist (i.e., painter or composer); and (5) presence of other people, when aesthetic experience occurs in social contexts. These factors were inspired by previous studies (Scherer and Zentner, 2001).

Another item focused on motivation, and participants were asked to rate the importance of five potential reasons in their aesthetic experience with painting or music: (1) mood management or relaxation; (2) experiencing new emotions, which are not typical of everyday life; (3) self-education; (4) sharing emotions with others; and (5) keeping company when one feels lonely. These types of motivation were also derived from previous literature (Lonsdale and North, 2011).

### Statistical analyses

The main analyses compared self-reported frequency of emotions, contributing factors and motivation for the two types of aesthetic experience: looking at painting and listening to music. Other analyses compared between participants with and without art education. Considering the unequal sizes of the two samples, as well as of the groups with and without art education, we used analysis of variance (ANOVA) with Welch's correction for unequal variance, which is a robust method to protect against type I errors while conserving power (Kohr and Games, 1974). In addition, we used the Bonferroni method to correct the threshold of statistical significance for each set of analyses, as follows: *p* ≤ 0.005 (0.05/9) for self-reported frequency of emotions; *p* ≤ 0.01 (0.05/5) for perceived contributing factors; and *p* ≤ 0.01 (0.05/5) for self-reported motivation. Effect sizes are reported as $\eta_{\text{P}}^{2}$, where an effect of 0.01 is small, one of 0.06 is medium, and one of 0.14 is large (Cohen, 1988). All analyses were run in SPSS.

## Results

### Painting and music-related emotions

**Figure 1:**
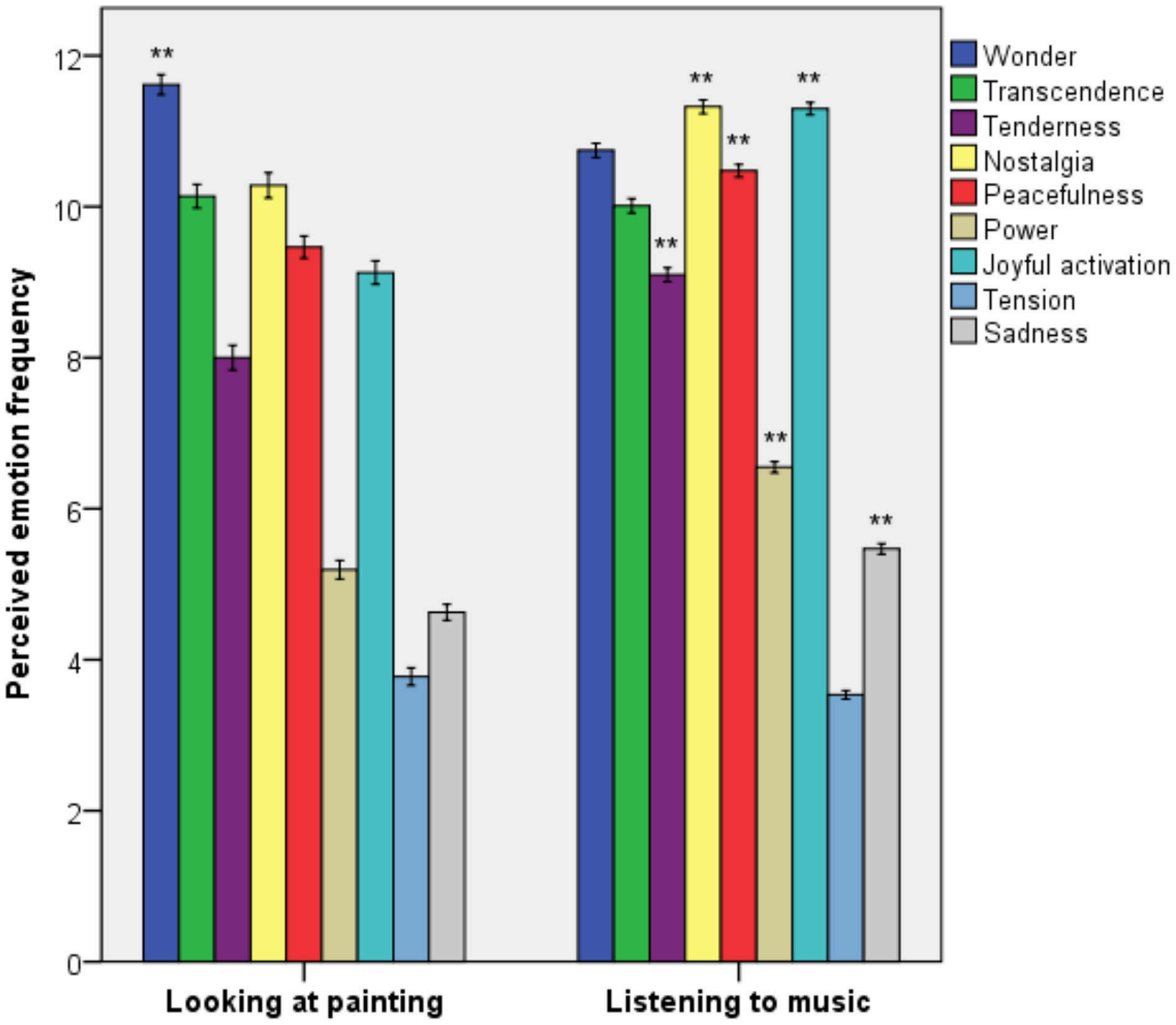
**Perceived frequency of emotions in the experience of looking at painting and listening to music**. Error bars indicate standard error of the mean. ^**^p < 0.01.

By comparing self-reported frequency of each emotion between samples (Figure 1), we found that those who described their experience of looking at painting reported higher frequencies of wonder compared to those who described their experience of listening to music [*F*_(1, 525.29)_ = 28.49, *p* < 0.001, $\eta_{\text{P}}^{2}$ = 0.03]. In contrast, the frequencies of tenderness [*F*_(1, 434.56)_ = 33.86, *p* < 0.001, $\eta_{\text{P}}^{2}$ = 0.04], nostalgia [*F*_(1, 419.57)_ = 30.09, *p* < 0.001, $\eta_{\text{P}}^{2}$ = 0.03], peacefulness [*F*_(1, 438.95)_ = 35.83, *p* < 0.001, $\eta_{\text{P}}^{2}$ = 0.04], power [*F*_(1, 447.32)_ = 89.75, *p* < 0.001, $\eta_{\text{P}}^{2}$ = 0.09], joyful activation [*F*_(1, 410.84)_ = 151.69, *p* < 0.001, $\eta_{\text{P}}^{2}$ = 0.15], and sadness [*F*_(1, 501.01)_ = 43.55, *p* < 0.001, $\eta_{\text{P}}^{2}$ = 0.04] were higher in relation to listening to music compared to looking at painting. The frequency of transcendence and tension were not different in the two samples.

The perceived similarity between art-related emotions and everyday emotions was also analyzed. Painting-related emotions (*M* = 3.25; *SD* = 0.99) were rated as significantly less similar to emotions in other everyday situations, compared to music-related emotions (*M* = 3.53; *SD* = 0.97): *F*_(1, 450.29)_ = 15.89, *p* < 0.001, $\eta_{\text{P}}^{2}$ = 0.02.

### Perception of contributing factors

**Figure 2:**
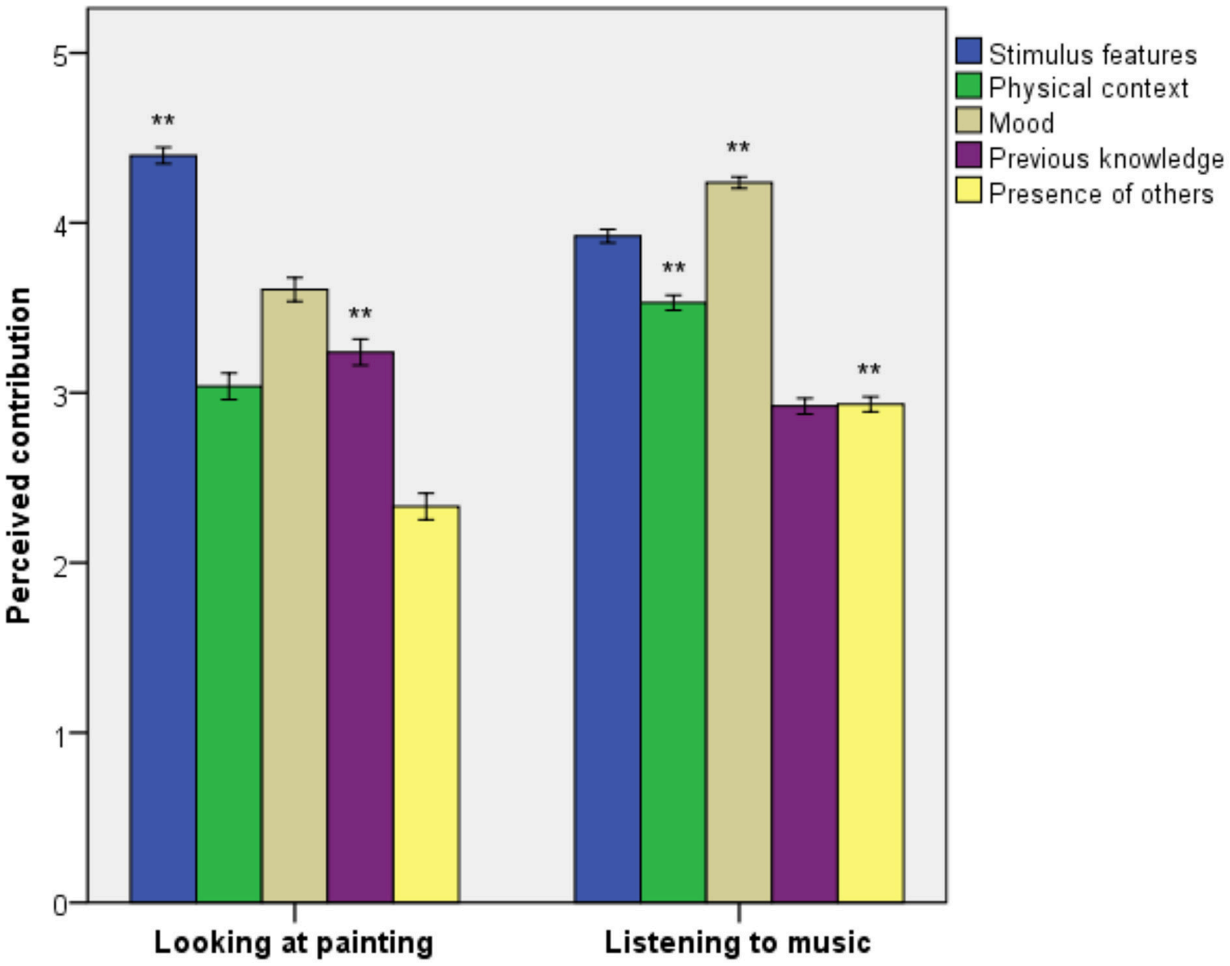
**Perception of factors contributing to painting and music-related emotions**. Error bars indicate standard error of the mean. ^**^p < 0.01.

Figure 2 shows the perceived contributions of several factors to art-related emotions. The contributions of stimulus features [*F*_(1, 624.81)_ = 56.85, *p* < 0.001, $\eta_{\text{P}}^{2}$ = 0.04] and previous knowledge [*F*_(1, 461.09)_ = 12.48, *p* < 0.001, $\eta_{\text{P}}^{2}$ = 0.01] were rated at higher levels for painting-related emotions, whereas the contributions of prior mood [*F*_(1, 384.60)_ = 65.93, *p* < 0.001, $\eta_{\text{P}}^{2}$ = 0.08], physical context [*F*_(1, 437.99)_ = 30.29, *p* < 0.001, $\eta_{\text{P}}^{2}$ = 0.03], and the presence of others [*F*_(1, 433.12)_ = 44.99, *p* < 0.001, $\eta_{\text{P}}^{2}$ = 0.05] were rated at higher levels for music-related emotions.

### Self-reported motivation

**Figure 3:**
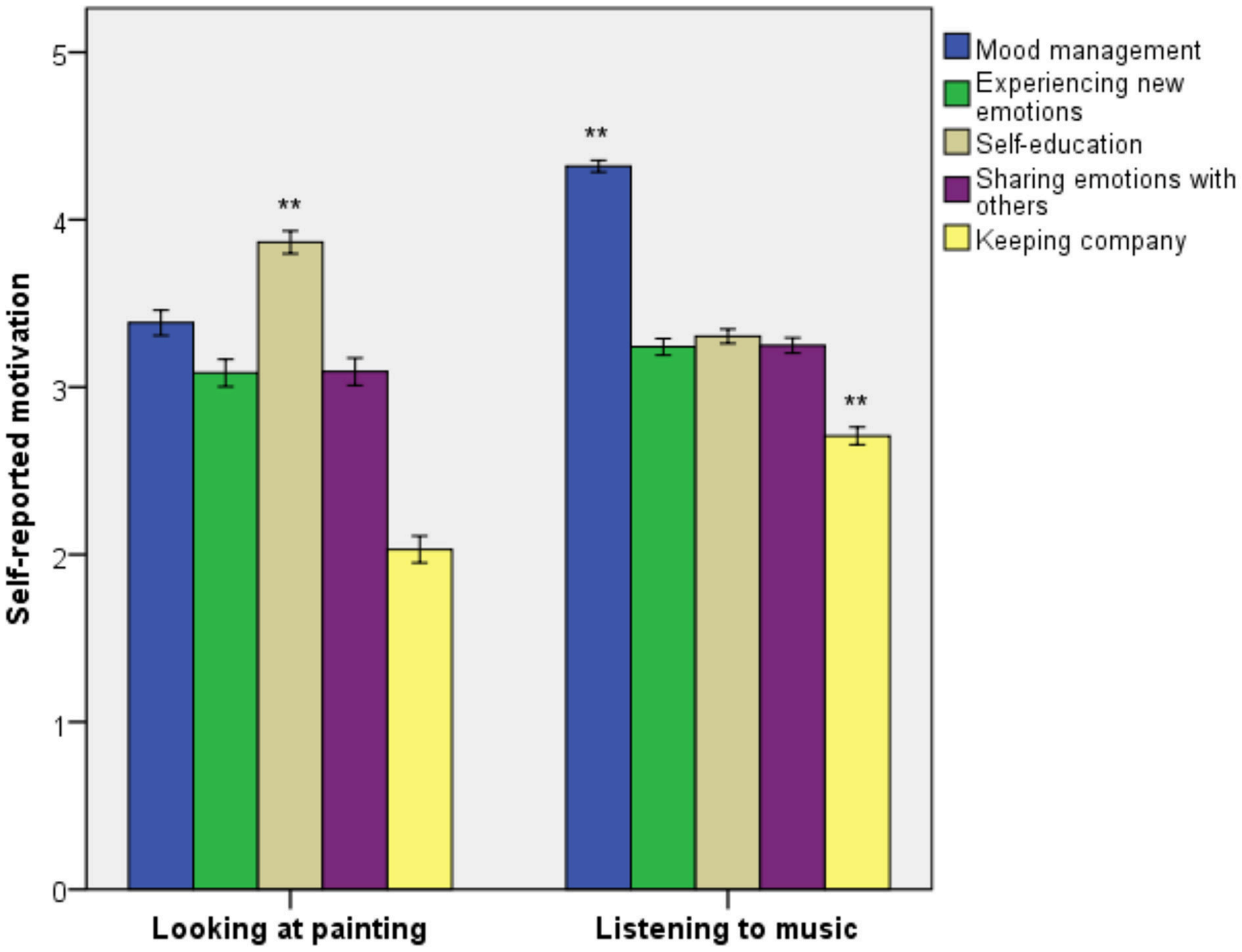
**Self-reported motivation for looking at painting and listening to music**. Error bars indicate standard error of the mean. ^**^p < 0.01.

Self-reported motivation was also compared between participants who described their experience of looking at painting and listening to music (Figure 3). Self-education was rated as more important for looking at painting [*F*_(1, 481.05)_ = 48.48, *p* < 0.001, $\eta_{\text{P}}^{2}$ = 0.05], whereas mood management [*F*_(1, 375.83)_ = 125.61, *p* < 0.001, $\eta_{\text{P}}^{2}$ = 0.14] and keeping company [*F*_(1, 506.15)_ = 50.21, *p* < 0.001, $\eta_{\text{P}}^{2}$ = 0.05] were rated as more important for music listening. Experiencing new emotions and sharing emotions with others were rated at comparable levels for looking at painting and music listening.

### Art education

There were 69 visual arts graduates in the sample that answered the painting survey, and 42 music graduates in the sample that answered the music survey. The majority of visual arts graduates reported knowledge about painting (99.65%), sculpture (55.07%), and art history (95.65%). The self-reported level of experience with painting [*t*_(258)_ = 7.53, *p* < 0.001, Cohen's d = 1.05), and the personal importance of painting [*t*_(258)_ = 5.04, *p* < 0.001, Cohen's *d* = 0.72] were significantly higher for visual art graduates compared to the other participants who filled in the painting survey. Similarly, most music graduates reported knowledge related to sight reading of music scores (92.86%), instrument playing (95.23%), and musicology (85.71%). The self-reported levels of experience with music [*t*_(51.74)_ = 7.56, *p* < 0.001, Cohen's *d* = 1.02] and the personal importance of music [*t*_(64.99)_ = 5.95, *p* < 0.001, Cohen's *d* = 0.65] were significantly higher for music graduates compared to the other participants who filled in the survey on listening to music.

Next, self-reported frequency of emotions, perception of contributing factors, and self-reported motivation for looking at painting and listening to music were compared between participants with and without art education in each sample (Table 2).

**Table 2:** **Perceived frequency of emotions in participants with and without arts education**.

		**Perceived frequency of emotions**									**Similarity to emotions in other situations**
		**Wonder**	**Transcendence**	**Tenderness**	**Nostalgia**	**Peacefulness**	**Power**	**Joyful activation**	**Sadness**	**Tension**	
Looking at painting	Visual arts graduates	11.46 ± 2.24	10.36 ± 2.56	8.3 ± 2.84	10.79 ± 2.57	9.81 ± 2.55	5.88 ± 2.17	10.17 ± 2.43	4.78 ± 2.01	4.26 ± 2.18	3.29 ± 0.98
	No formal visual arts education	11.67 ± 2.12	10.05 ± 2.52	7.89 ± 2.57	10.09 ± 2.72	9.34 ± 2.28	4.94 ± 1.86	8.74 ± 2.44	4.57 ± 1.59	3.6 ± 1.72	3.23 ± 1
Listening to music	Music graduates	10.9 ± 2.1	10.5 ± 2.57	8.78 ± 2.29	11.54 ± 2.59	10.09 ± 2.03	6.59 ± 1.86	10.85 ± 2.24	5.42 ± 1.66	3.57 ± 1.43	3.36 ± 1.2
	No formal music education	10.73 ± 2.50	9.98 ± 2.53	9.11 ± 2.48	11.3 ± 2.4	10.49 ± 2.24	6.54 ± 1.93	11.32 ± 2.18	5.47 ± 1.89	3.53 ± 1.51	3.54 ± 0.95

*Values in cells are means and standard deviations*.

Participants with visual arts education reported significantly higher frequencies of power [*F*_(1, 106.04)_ = 10.18, *p* = 0.002, $\eta_{\text{P}}^{2}$ = 0.04] and joyful activation [*F*_(1, 120.54)_ = 17.32, *p* < 0.001, $\eta_{\text{P}}^{2}$ = 0.06] in their experience with painting, in comparison to participants without visual arts education. Frequencies of the other painting-related emotions were not significantly different between those with and without visual arts education. Self-reported frequencies of all music-related emotions were similar in participants with and without music education.

Perceived similarity between art-related (i.e., painting or music) and everyday emotions was not significantly different in participants with and without art education (i.e., visual arts education or music education).

Both participants with visual arts education [*F*_(1, 122.63)_ = 6.81, *p* = 0.010, $\eta_{\text{P}}^{2}$ = 0.03] and those with music education [*F*_(1, 46.04)_ = 23.91, *p* < 0.001, $\eta_{\text{P}}^{2}$ = 0.03] rated the contribution of previous knowledge to painting-related emotions and music-related emotions, respectively, as more important, in comparison to participants without art education (Table 3).

**Table 3:** **Perception of factors contributing to art-related emotions in participants with and without arts education**.

		**Perception of contributing factors**				
		**Stimulus features**	**Physical context**	**Mood**	**Previous knowledge**	**Presence of other people**
Looking at painting	Visual arts graduates	4.51 ± 0.76	3.35 ± 1.27	3.55 ± 1.15	3.57 ± 1.2	2.57 ± 1.30
	No formal visual arts education	4.36 ± 0.78	2.93 ± 1.22	3.63 ± 1.11	3.12 ± 1.23	2.25 ± 1.23
Listening to music	Music graduates	4.29 ± 0.94	3.4 ± 1.14	4.21 ± 0.84	3.83 ± 1.24	2.74 ± 1.08
	No formal music education	3.9 ± 1.06	3.54 ± 1.18	4.24 ± 0.89	2.86 ± 1.21	2.94 ± 1.17

*Values in cells are means and standard deviations*.

There were no significant differences related to art education in self-reported motivation for looking at painting or listening to music (Table 4).

**Table 4:** **Self-reported motivation for looking at painting and listening to music in participants with and without arts education**.

		**Motivation**				
		**Mood management**	**Experiencing new emotions**	**Self-education**	**Sharing emotions with others**	**Keeping company**
Looking at painting	Visual arts graduates	3.43 ± 1.27	3.22 ± 1.32	4.14 ± 1.01	3.23 ± 1.33	2.13 ± 1.32
	No formal visual arts education	3.37 ± 1.2	3.04 ± 1.31	3.76 ± 1.11	3.04 ± 1.31	1.99 ± 1.26
Listening to music	Music graduates	3.86 ± 1.29	3.62 ± 1.39	3.64 ± 1.24	3.38 ± 1.36	2.6 ± 1.43
	No formal music education	4.35 ± 0.9	3.22 ± 1.31	3.28 ± 1.14	3.24 ± 1.22	2.72 ± 1.42

*Values in cells are means and standard deviations*.

## Discussion

In this study, participants answered surveys on their experience of looking at painting and listening to music. The main aims were to compare between perceptions regarding frequency of emotions, contribution of several factors to art-related emotions, and motivation for these two types of aesthetic experience. In addition, we examined the influence of art education on these dimensions.

Previous studies identified emotions that are commonly experienced by music listeners (Zentner et al., 2008). Aesthetic emotions such as awe (Shiota et al., 2007) and other positive and negative emotions that occur in various everyday situations (Silvia, 2011) have also been described in the experience of looking at painting. These studies suggested that looking at painting and listening to music are associated with blends of different types of emotions. However, no study has yet compared the relative frequency of different emotions in these two types of aesthetic experience. The present results indicate that wonder may be more frequently experienced while looking at painting rather than while listening to music. In addition, the experience of looking at painting may be associated with relatively lower frequency of vitality-related emotions (Zentner et al., 2008) such as joyful activation and power. These two emotions were much more frequently (i.e., large or medium effect size) reported in relation to listening to music, which suggests that “vitality” may best distinguish emotional responses to music and painting. Other emotions (i.e., tenderness, nostalgia, peacefulness, sadness) were also more frequently reported in the experience of listening to music compared to looking at painting, but to a lesser degree, that is, with small effect sizes.

Painting-related emotions were perceived as less similar to emotions experienced in other everyday life situations compared to music-related emotions. This perception may be connected to the relatively higher frequency of wonder associated with looking at painting, considering that this emotion is experienced in limited contexts (e.g., contemplation of artworks or nature scenes; Shiota et al., 2007) that create the sensation of being in the presence of something greater than oneself (Keltner and Haidt, 2003). The reduced vitality of emotions associated with looking at painting may also contribute to the impression that they are different from emotional experience in general.

These results also indicate differences in the perception of factors that may contribute to art-related emotions. Participants rated stimulus features and previous knowledge as making more important contributions to emotional responses to painting than to music. These impressions are in line with theories (Berlyne, 1974) and experimental evidence (Jacobsen and Höfel, 2002; Leder et al., 2012; Ticini et al., 2014; McLean et al., 2015; Topolinski et al., 2015) that support the relation between perceptual features of paintings and their emotional impact. The present observations do not exclude the contribution of these factors to music-induced emotions, which is well documented in the literature (Gabrielsson and Lindstrom, 2001; Gomez and Danuser, 2007), but merely suggest that people perceive them as weighing more in the experience of looking at painting. In addition, the perception that previous knowledge plays an important role in painting-related emotions was corroborated by another observation in this study (see below), namely that the frequency of certain painting-related emotions was higher in visual art graduates, who reported higher levels of art knowledge. In a complementary way, the influence of prior mood, physical context, and the presence of other people were rated as more important in relation to music-induced emotions. These subjective evaluations are also in line with previous evidence showing that indeed, both mood prior to music exposure, whether in laboratory (Hunter et al., 2011; Vuoskoski and Eerola, 2011b) or concert hall (Vuoskoski and Eerola, 2011b; Baltes and Miu, 2014), and the presence of others, particularly close persons (Juslin et al., 2008; Liljestrom et al., 2013), influence emotional responses to music.

Experiences of looking at painting and listening to music were also differentiated by self-reported motivation. Relatively more participants reported that self-education motivated them to look at painting. In addition, relatively more participants reported that mood repair and keeping company drove their experience of listening to music. These motivational differences may be supported by many factors, including the wider accessibility of music on portable devices, which may increase its use for everyday life needs such as mood repair (Lonsdale and North, 2011), and the relatively higher vitality of emotional responses to music, which may contribute to increasing function in everyday life. Pending on replication of these results, future research could examine why people use the experience of looking at painting and listening to music for relatively different reasons.

Visual arts graduates reported higher frequencies of power and joyful activation in their experience of looking at painting. Considering that these emotions had the lowest frequencies in the overall sample that answered the painting survey, this indicates that visual arts formal training has a significant impact on emotional responses to painting and may specifically enhance vitality-related emotions. In contrast, music formal training had no significant effect on the frequency of music-related emotions, which is in line with previous evidence (Bigand and Poulin-Charronnat, 2006; Baltes et al., 2012). These findings suggest that painting-related emotions may involve knowledge-driven or top-down information processing to a larger extent than music-related emotions. However, both visual arts and music graduates rated the contribution of previous knowledge (e.g., information about artwork and artist) to emotional responses at higher levels than participants without formal art training. No differences in motivation for looking at painting and listening to music were linked to formal art education. Given that art graduates reported increased levels of art-related knowledge—although note that this type of knowledge was not limited to those with formal training—, as well as increased experience with and personal importance of art, these differences may have driven the present observations on the influence of formal art training.

This study has at least two main limitations. First, being based on surveys, these findings describe how art-related experience is perceived by people, and may thus be subjectively biased. For instance, all art graduates reported that increased levels of art knowledge would enhance art-related emotions, but only visual arts education seemed to influence emotional responses to painting. Second, we assessed emotional experience using a scale that focuses on emotions which are common in the experience of listening to music. There is no similar scale for painting-related emotions, so the only available options for this study were measures focused on music-induced emotions such as GEMS (Shiota et al., 2007) and general measures such as PANAS (Watson and Clark, 1994). We chose the former option considering that GEMS, which was developed through a factorial approach based on self-reported experience of music listeners (Zentner et al., 2008), may offer a more specific assessment of aesthetic emotions, leaving out emotions that are not representative for the experience of music listening and may be equally unrepresentative for the experience of looking at painting. Previous studies suggested some similarities between emotional responses to painting and music (Shiota et al., 2007). In addition, GEMS and PANAS partially overlap, with emotions like wonder, power, joyful activation, tension, and sadness from the former scale paralleling emotions like serenity, self-assurance, joviality, hostility, and sadness from the latter scale. Notwithstanding these reasons in favor of our approach, it is possible that we did not assess emotions that are more specific to looking at painting and are not covered by GEMS. For instance, recent studies identified so-called “knowledge emotions” such as surprise, interest and confusion in the experience of looking at painting (Silvia, 2011). Therefore, the specificity of painting-related emotions may have been underestimated in this study. Future research may identify other specific aspects of emotional responses to painting.

In conclusion, our results highlighted multiple differences in the perceived qualities of looking at painting and listening to music: emotional responses to painting may be characterized by higher levels of wonder and lower vitality, and are perceived as less similar to emotions in other everyday life situations, compared to music-induced emotions; people outweigh the contributions of stimulus features and previous knowledge in relation to emotional responses to painting, and the contributions of prior mood, physical context, and the presence of others in relation to emotional responses to music; looking at painting is driven by self-education motivation, whereas listening to music is associated with emotional and social motivation; and formal art training influences emotional responses to painting (e.g., by increasing vitality), but not to music, which suggests that the former may depend more on knowledge-driven information processing. We hope this study will encourage the integration of theories and approaches in research on painting and music, which have largely developed in parallel until now, and stimulate future research that could give a more detailed perspective on common and specific aspects of aesthetic experiences with different forms of art.

### Conflict of interest statement

The authors declare that the research was conducted in the absence of any commercial or financial relationships that could be construed as a potential conflict of interest.
